# Molecular identifiers of the evolutionarily conserved titin pseudokinase

**DOI:** 10.1042/BCJ20253442

**Published:** 2026-01-07

**Authors:** Till Dorendorf, Peter Gravenhorst, Olga Mayans

**Affiliations:** 1 Department of Biology, University of Konstanz, Universitätsstraße 10, Konstanz, 78457, Germany; 2 Konstanz Research School of Chemical Biology (KoRS-CB), University of Konstanz, Universitätsstraße 10, 78457 Konstanz, Germany

**Keywords:** molecular evolution, pseudokinase, sequence analysis, titin kinase, titin protein classification, X-ray crystallography

## Abstract

Titin kinase (TK) is an enigmatic pseudokinase specific to the striated muscle of vertebrates. Embedded within the contractile sarcomere and flanked by extensible regulatory tails, TK is thought to act as a mechanoreceptor that senses mechanical signals arising from muscle function. Studies on TK so far have focused narrowly on the human representative, whose phosphotransfer activity remains questioned. To investigate whether the pseudokinase character is a hallmark of TK, we studied sequences of distantly evolved fish representatives and rationalized conservation patterns by solving the crystal structure of TK from medaka (isoform *b*). We find that sequence deviations in functional motifs involved in ATP and magnesium binding, respectively θxK (θ: bulky hydrophobic residue) and EFG, are evolutionarily conserved in TK. Beyond the kinase domain, N- and C-terminal flanking tails show remarkable structural similarity across orthologues, even though sequence conservation is limited to individual residues and short motifs: a YD-motif in the N-terminal tail; a [R/K]H[R/K]RYY sequence, a R-7x-R motif and position -2 of the latter in the C-terminal tail. Motifs in the C-terminal tail consistently covary with the divergent functional motifs of TK, being part of its pseudokinase signature. Contrary with these general features, the putatively inhibitory interaction of the catalytic aspartate with a tyrosine from loop P+1 is primarily confined to mammals. Finally, based on sequence clustering analysis, we identify TK subgroupings and propose a classification of titin genes from fish into *a* and *b* isoforms (*ttna* and *ttnb*) that can assist future studies. A curated genomic annotation is provided.

## Introduction

The giant protein titin (~3–4 MDa) plays a central role in generating, sensing and transmitting passive force in the muscle sarcomere [1,2]. It is a multi-domain, elastic protein primarily composed of serially linked immunoglobulin and fibronectin domains, which spans the half-length of the sarcomere, from Z-disc to M-line (>1 µm in length) [3]. A single signalling domain is found in titin, a kinase domain (titin kinase; TK) near the C-terminus of the protein in the sarcomeric M-line. The function of TK has remained enigmatic ever since its discovery over 30 years ago [4]. However, being embedded within the contractile sarcomere, it is regarded as a prime mechanoreceptor candidate to sense and transduce the mechanical signals arising from muscle function, communicating sarcomere demands to other cellular compartments.

TK mechanosensing is thought to be mediated by extensible tail sequences that flank the kinase N- and C-terminally (N-terminal linker: NL; C-terminal regulatory domain: CRD). Structural characterization of human TK showed that the NL and CRD flanking tails pack tightly against the kinase domain, blocking the interlobular hinge and the active site, respectively [5,6]. In invertebrates, the close homologue twitchin kinase (TwcK) also displays this same architecture, as revealed by representatives from *Caenorhabditis elegans* (*ce*TwcK) [7–9] and *Aplysia californica* (*ac*TwcK) [8] (40% and 41% sequence identity to human TK, respectively). In *ce*TwcK, the tails have been shown to inhibit phosphotransfer catalysis [8–10], so that kinase activation requires their removal from the kinase surface. It is postulated that in TK and TwcK, tail removal is elicited by mechanical force, with the pulling forces that develop in the sarcomere during muscle function causing the tails to unravel, exposing the kinase active site [6,9,11,12]. Support to the physiological relevance of this mechanism was brought by a study that monitored fluorescence resonance energy transfer (FRET) in live, freely swimming *C. elegans* to reveal conformational changes in *ce*TwcK during muscle activity [13].

While tail removal is expected to trigger kinase-mediated force-dependent signalling pathways in the cell, it is the case that TK is a pseudokinase of unclear phosphotransfer output [14]. In TK, two catalytic motifs are altered when compared with canonical kinases: MAK (instead of the canonical AxK sequence) in the ATP-binding pocket and an EFG motif (instead of DFG) in the magnesium binding site [14,15]. The AxK motif in strand-β3 of the kinase fold contains the essential catalytic lysine that binds ATP in a position compatible with the productive transfer of the γ-phosphate and, in addition, forms a salt bridge with a conserved glutamate from helix αC that yields an active kinase conformation [16]. In active kinases, small hydrophobic residues occupy position 1 of the AxK motif (typically alanine or valine), as this residue forms the bottom of the cavity that accommodates the nitrogenated base in ATP [16]. In TK, the bulkier methionine residue at this position is speculated to sterically conflict ATP binding [14]. In the DFG motif, aspartate chelates the magnesium ion required for γ-phosphate transfer. Its substitution for glutamate drastically reduces phosphotransfer activity in canonical kinases [14,17,18]. It is expected that also in TK, the presence of a glutamate conflicts activity. In agreement with these deductions, validated substrates and phosphotransfer-dependent pathways are yet to be identified for TK.

Even when catalytically inactive, pseudokinases still function as crucial signalling regulators. They act as scaffolds to build protein complexes, serve as allosteric switches to activate or inhibit other kinases, act as competitive inhibitors and control protein localization in the cell [19]. In this regard, the scaffolding functions of TK are better established. TK associates with the E3 ubiquitin ligases MURF1 [6,14] and MuRF2 [20] and the autophagy receptors p62 and Nbr1 that promote the autophagy of poly-ubiquitinated proteins [6,20]. While the functional outcomes of such scaffolding are not yet fully understood, it has been proposed that the release of MuRF2 from its TK locus in inactive muscle cells leads to suppression of the nuclear serum response factor, which in turn hinders the transcription of anabolic genes; this is in good alignment with mechanical inactivity [20]. However, a more recent study [6] found that ubiquitination of the TK locus by MuRF1 led to its targeting by Nbr1 and p62 that associate with the ubiquitin moiety, presumably being functionally linked to sarcomere loss processes. The study proposed that the extension of the kinase tails resulting from muscle activity decreased TK targeting by MuRF E3 ubiquitin ligases, reducing ubiquitination levels and Nbr1 and p62 recruitment, allegedly aiding sarcomere preservation. Taken together, these findings have led to regarding TK as a mechanosensory scaffold with functional links to protein turn-over pathways and gene regulation.

Studies of TK to date have focused narrowly on the human representative, so that it is unknown to which extent the function of this kinase is conserved across vertebrates. To gain an insight into TK conservation, in this work we have studied sequences from distantly related fish representatives, the most ancestral class of vertebrate. Fish constitute a rich source of diversified protein sequences, having developed over long evolutionary distances and containing duplicate gene variants dating back to the teleost-genome duplication that occurred 267–286 million years ago [21]. Zebrafish (*Danio rerio*) and medaka (*Oryzias latipes*) are two teleostei model fish organisms that represent different phylogenetic groups. Medaka belongs to the more recently emerged monophyletic clade of the neoteleostei, whereas zebrafish branched off earlier in the teleost evolution. Among fish, the titin copies of zebrafish are best studied. These two titin isoforms (ttna and ttnb) have been attributed different functions. Isoform ttna is highly expressed in cardiac muscle (ratio of 2:1 ttna:ttnb), where it is essential for sarcomere formation, whereas isoform ttnb is dispensable in this muscle type [22]. In contrast, the expression ratios of ttna and ttnb in skeletal muscle sarcomeres are similar and both isoforms are required for sarcomere formation [22,23]. Similar to zebrafish, medaka also contains two copies of the titin gene, but they are uncharacterized. Guided by these model systems, we have performed a sequence analysis of titin isoforms in fish and compared results with mammalian representatives. Complementing this analysis, we have elucidated the crystal structure of TK from the ttnb isoform of *Oryzias latipes* (medTK*b*) that allows us to rationalize sequence conservation patterns. This analysis has led us to identify the evolutionarily conserved molecular determinants of TK, reveals the existence of TK subgroups and facilitates the understanding of TK-related sarcomeric kinases.

## Results

### Comparing TK sequences from fish reveals distinct subtypes

**Figure 1:**
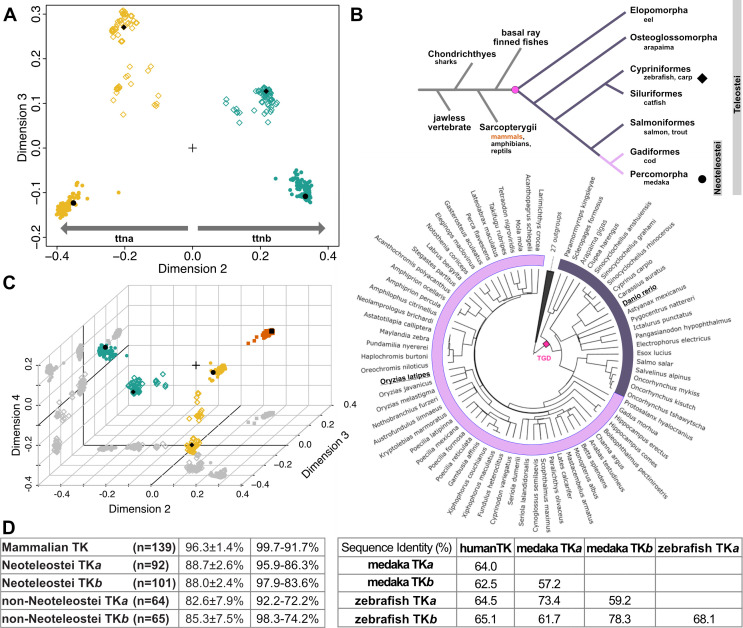
PaSiMap-based comparison of titin kinase sequences. **A.** Clustering of TK sequences from fish visualized in a 2D-vector map from PaSiMap [24]. The sequences analysed encompass the NL segment, the TK domain and the CRD-tail. Four sequence clusters are identified. Yellow and teal colours indicate TK isoforms *a* and *b*, respectively. Fish groups are indicated by diamond (non-neoteleostei) and circular (neoteleostei) data points. TK sequences from the model organisms zebrafish (*Danio rerio*; ◆) and medaka (*Oryzias latipes*; ●) are indicated; **B**. Simplified representation (*upper panel*) of vertebrate evolution including the branching of the mammalian lineage and a basic phylogenetic tree of fish. A more comprehensive fish phylogenetic representation is also shown (*lower panel*) (reproduced and colour adapted from [26]). In both representations, the point of teleost-genome-duplication is indicated as a dot in magenta, with neoteleostei being shown in pink and older fish in aubergine; **C**. PaSiMap clustering of fish and mammalian TK sequences. Fish colouring is as in A., mammalian TKs are shown as brown squares (human TK is indicated; ■). To aid 3D-visualization, data point projections on the axial planes are shown as shadows (grey); **D**. Matrix of sequence identities (expressed in percentage) within PaSiMap sequence clusters corresponding to animal groups (*left*) and across representatives of main groups (*right*). Within a same cluster, sequence identity is calculated as average sequence identity ± standard deviation and range of values is given; number of sequences in each group is stated.

In order to undertake a comparative study, TK sequences from fish were collected by performing a similarity search in Blast (https://blast.ncbi.nlm.nih.gov) using human TK as query. Upon curation, the search yielded 320 sequences from teleost fish (provided in Supplementary Table S1). Aiming to reveal sequence groups sharing close similarity within the set, the sequences were subjected to clustering analysis using PaSiMap, an algorithm that transforms pairwise similarity values into correlation coefficients and projects them into a multi-dimensional space [24,25]. Each sequence is graphically depicted as a vector in space from the co-ordinate origin. PaSiMap analysis caused the fish TK sequences to segregate into four clusters (the interactive 3D-vector map from PaSiMap is provided as Supplementary Table S2). A 2D representation of dimension 2 vs. dimension 3 of the resulting vector map (Figure 1A) shows that each sequence cluster localizes to a quadrant of the co-ordinate system. Dimension 2 separates the sequences according to TK isoforms, *a* and *b* (i.e. TK copies that emerged through gene duplication in the same organism localize to different clusters). TK from zebrafish ttna localizes to the cluster located below the axis origin (0), whereas TK from zebrafish ttnb is found in the cluster above 0. Using as reference these annotated isoforms from zebrafish, we extend here the isoform *a* and *b* nomenclature to the rest of TK sequences in the corresponding clusters. Aiming to reveal whether the TK classification is representative of full-length titin and, thus, whether it could be employed in titin gene annotation, we performed a further PaSiMap cluster analysis on selected full-length titin sequences from fish. Specifically, the analysis included both titin isoforms from medaka, ocellaris clownfish (*Amphiprion ocellaris*) and tuna (*Thunnus albacares*) from the neoteleostei clad and zebrafish (*Danio rerio*), goldfish (*Carassius auratus*) and yellowhead catfish (*Tachysurus fulvidraco*) as non-neoteleostei (Supplementary Figure S1). The analysis reproduced the results obtained from single TK domains, leading us to conclude that TK is a valid proxy for the classification of titin genes in fish. Thus, we propose here adopting the nomenclature *ttna* and *ttnb* for titin genes from the 320 fish species studied in this work. The new gene annotation is given in Supplementary Table S1.

In the PaSiMap vector map, sequence segregation in dimension 3 correlates with fish group (Figure 1B); namely, all sequences from neoteleostei (e.g. medaka, ocellaris clownfish and tuna) have values below zero, while sequences from evolutionarily older fish (e.g. zebrafish, goldfish or yellowhead catfish) have values above zero. This indicates that these fish groups can also be identified and catalogued through the analysis of TK. Importantly, PaSiMap places isoform type in dimension 2 and fish group in dimension 3. As the order of dimensions corresponds to the prominence of the sequence divergences, this signifies that differences between TK isoforms in the same organism are larger than differences within the same isoform across fish groups. This deduction agrees with the order of evolutionary events in fish, and therefore, the evolutionary distances of the sequences studied (Figure 1B).

To investigate how the TK*a* and TK*b* domains from fish relate to the better characterized human TK, we extended the PaSiMap analysis to mammalian sequences. The original Blast search also yielded 139 sequences of mammalian TKs, which were then added to the fish counterparts and subjected to clustering. The resulting vector map (Figure 1C; interactive PaSiMap 3D-vector map provided as S upplementary Table S3) segregated mammalian TK sequences into an additional fifth cluster, suggesting that fish TK*a*, TK*b* and mammalian TK form distinct groups without mutual overlap. To analyse sequence proximity within individual clusters and across clusters, sequence identity levels were quantitated (Figure 1D). Within clusters, sequence identity is high in all cases, but mammalian TK sequences share the highest identity levels (96.3%) even though the sequences originate from phylogenetically distant animals such as the egg-laying mammal *Tachyglossus aculeatus*, the marsupial *Monodelphis domestica* or the narwhal. Fish TK sequences also show high conservation levels within the clusters, but the older non-neoteleostei group is more diversified, as could be expected. To exemplify sequence identity across clusters, we compared human, medaka and zebrafish TK representatives (Figure 1D **, right**). This analysis yielded values ranging from 57.2% to 78.3% and confirmed that conservation is higher within each isoform type, i.e., isoforms *a* share higher identity across all fish groups than to isoforms *b* within the same organism*,* which is also true for isoforms *b*. This agrees excellently with deductions drawn from examining PaSiMap dimensionality (above).

### Structural characterization of medaka TK*b* reveals a conserved fold

**Figure 2:**
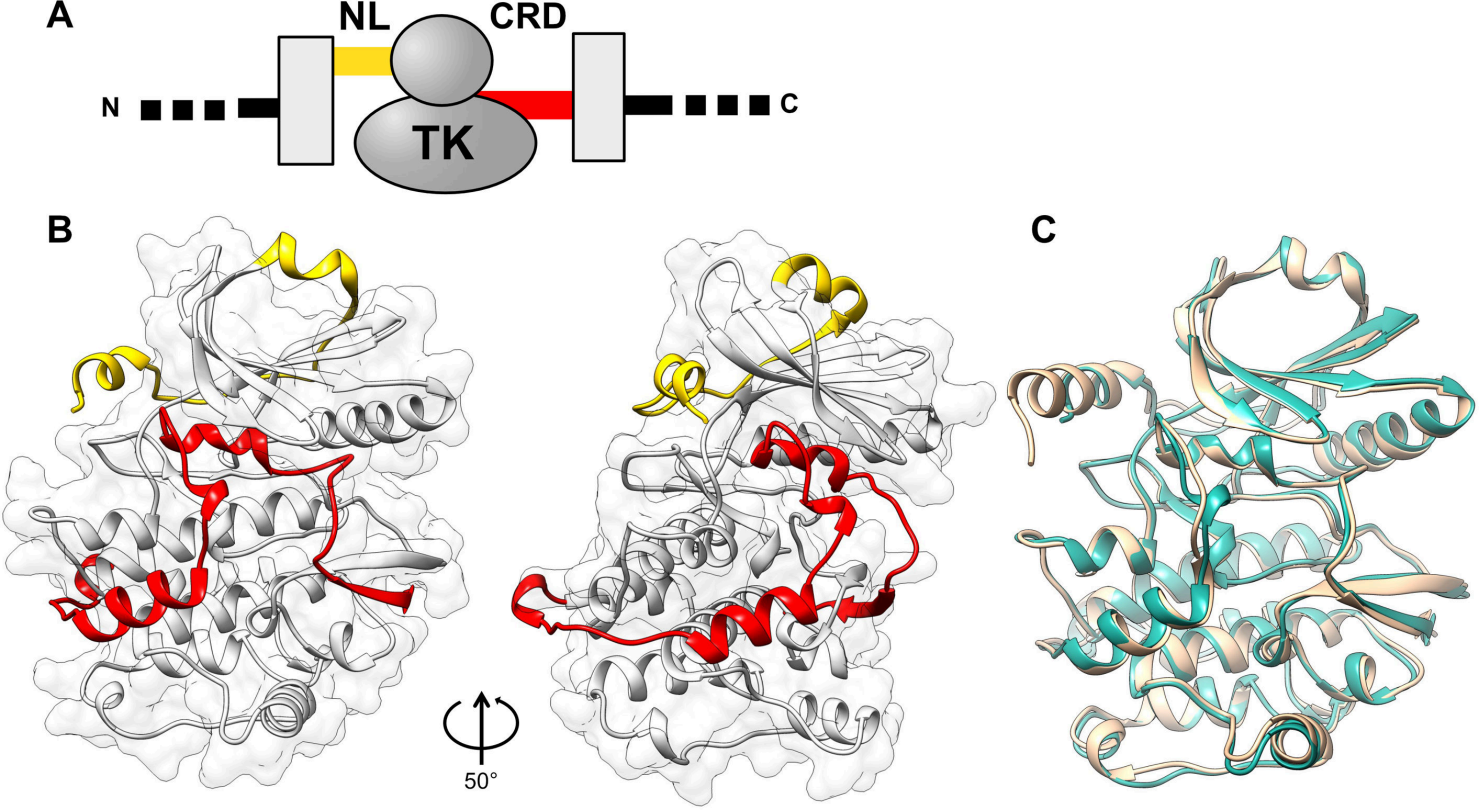
Crystal structure of medaka TK*b*. **A.** Domain composition of the kinase region of titin proteins. The kinase domain (TK) is flanked N-terminally by a linker sequence (NL, yellow) that joins it to a preceding fibronectin domain. C-terminally, TK is flanked by a regulatory tail extension (CRD, red), which is followed by an immunoglobulin domain. Neighbouring fibronectin and immunoglobulin domains are shown as grey boxes; **B**. Crystal structure of medaka TK*b* shown in two orientations (approx. ~50° rotation around an axis contained in the plane of the image). Colour code is as in A. The surface outline corresponds to the kinase domain; **C**. Superimposition of medaka TK*b* (teal) with the corresponding segment of human TK (beige) extracted from the multi-domain structure in PDB entry 6YGN [6]; (RMSD_Cα_ = 1.10 Å for all 345 overlapping C_α_-atoms; calculated using UCSD Chimera; https://www.cgl.ucsf.edu/chimera).

To gain a better understanding of the molecular diversity of TK kinases, we have elucidated the crystal structure of the most divergent representative from a fish model organism, TK isoform *b* from medaka (medTK*b*), to 2.1 Å resolution (Table 1; Figure 2A and B). The construct comprised the NL-kinase-CRD regions. The crystal form obtained in this study contained two molecular copies in the asymmetric unit, which were essentially identical (RMSD_Cα_ = 0.284 Å for all protein residues). The crystal structure of medTK*b* closely resembles that of human TK [5,6,14] (RMSD_Cα_ = 1.10 Å), with which it shares 62.5% sequence identity (Figure 2C). Remarkably, the highly conserved 3D fold of TK extends to its NL and CRD flanking tails even though sequence conservation in the latter is low and mostly confined to single residues or short motifs (described below). Only a minor deviation of the fold can be observed, localized to the 8-residue loop region between helices αR1 and αR2 in the CRD tail. In conclusion, the 3D fold of medTK*b* closely agrees with that of the well-studied human orthologue.

**Table 1:** X-ray data statistics and model parameters

	medTKb
PDB code	9QJ2
Space group	P21
Cell dimensions: a,b,c (Å)	61.74, 56.08, 120.25
Cell angle: ⍺, β, ɣ (°)	90.00, 104.28, 90.00
Copies in ASU	2
**Data processing**	
Beamline	DESY beamline P14
Detector	EIGER2 16M
Wavelength (Å)	0.9763
Resolution (Å)	30–2.1 (2.15–2.1)^1^
No. reflections	46,545 (3175)^1^
Rsym(I) (%)	12.2 (258.2)^1^
<I/σ(I)>	10.55 (0.91)^1^
CC1/2 (%)	98.8 (37.0)^1^
Completeness (%)	99.1 (99.2)^1^
Multiplicity	6.88 (7.04)^1^
**Model refinement**	
No. working/free reflections	46535 / 1427
Rwork/Rfree (%)	19.02 / 22.72
No. residues / atoms protein	688 / 5516
NCS R.m.s.d (Å)	0.284
No. atoms solvent	215 + 11 x EDO^2^
R.m.s.d. bond length (Å)	0.007
R.m.s.d. angles (°)	0.900
Ramachandran plot	
Favoured/disallowed (%)	98.68/0.0

1 values for the highest resolution shell

2 EDO: 1,2-ethanediol

### Conservation of pseudokinase features in the TK domain

**Figure 3:**
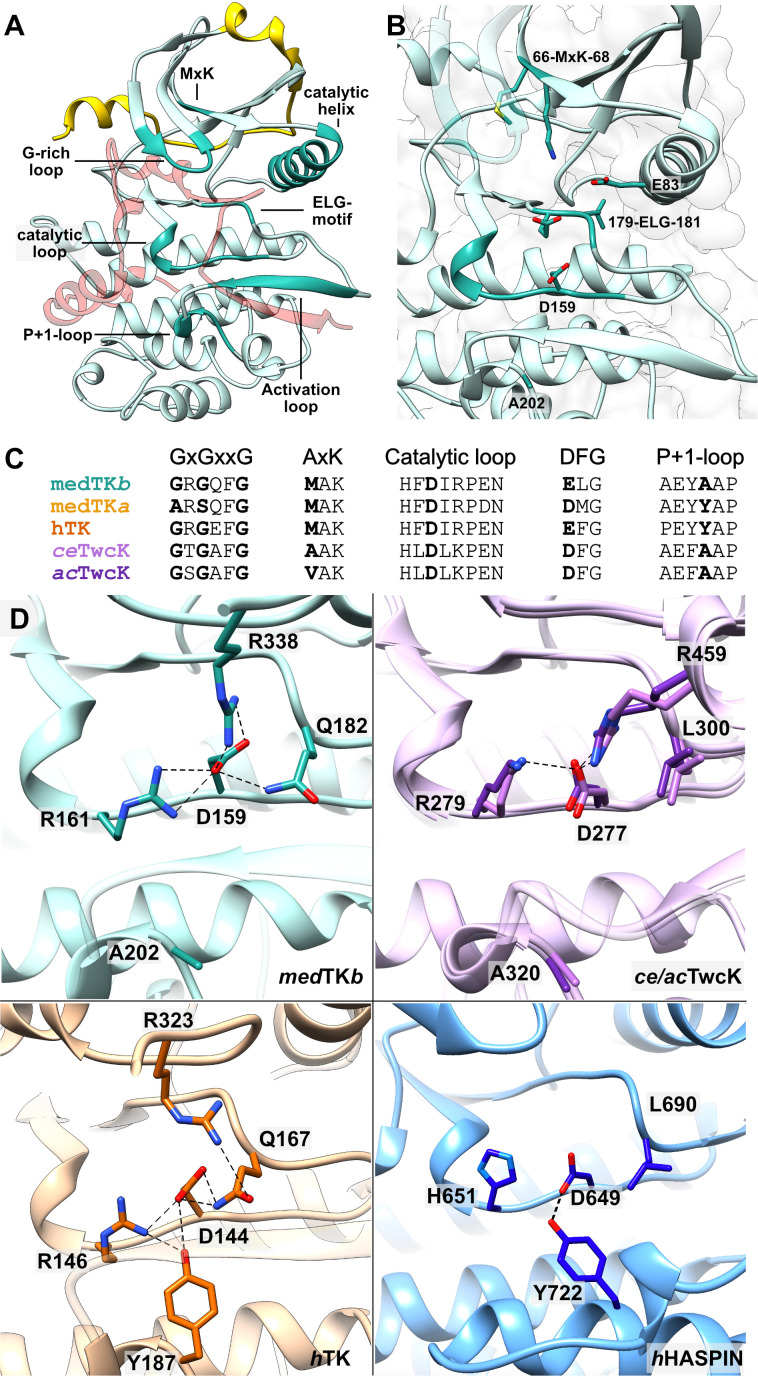
Catalytic features of medTK*b*. **A.** Crystal structure of medTK*b* with functionally relevant features highlighted in turquoise and annotated; namely, the ATP-binding glycine-rich loop, the MAK motif in strand β3, the metal cofactor chelating ELG motif, the activation loop, the *P*+1 loop that harbours the putatively inhibitory tyrosine in human TK, and the catalytic loop that harbours the catalytic aspartate. The NL linker is shown in yellow and the CRD tail in transparent red; **B**. Active site of medTK*b*; **C**. Sequence alignment of selected functional motifs in TK from medaka isoforms, human TK, and TwcK of known 3D-structure; **D**. Comparative overview of the interactions sustained by the catalytic aspartate in representative kinases. Shown are medTK*b* (this study), *C. elegans (ce;* lilac) and *A. californica (ac;* purple) TwcK (PDB 3UTO [9] and 1KOB [8], respectively) (residue numbering belongs to *ce*TwcK), human TK (PDB 4JNW [14]), and human HASPIN kinase (PDB 2WB8 [34]). For visual clarity, the CRD is excluded from the images of TK and TwcK structures. Hydrogen bonds are shown as dotted lines.

Catalytic readiness in kinases is achieved by finely choreographed conformational changes that collectively make the kinase transition from an ‘off’ to an ‘on’ state [27,28]. The catalytically ready state is defined by *i*) the position of helix αC, which allows the formation of a salt bridge between its strictly conserved glutamate and the invariant lysine from strand β3 in the AxK motif; *ii*) an open conformation of the activation loop that allows access to the active site; *iii*) the assembly of two spines (R(regulatory)-spine and C(catalytic)-spine) of stacked hydrophobic residues that define the arrangement of the kinase lobes; and *iv*) the ‘in’ arrangement of a conserved DFG motif, where the F residue is oriented towards the interior of the kinase completing the R-spine and the D residue, involved in the co-ordination of the Mg^2+^ cofactor, is accessible in the active cleft. These conformational changes in kinases are commonly induced by the binding of ATP. However, with the exception of feature *i),* the hallmarks of the active state are already displayed by the autoinhibited TK domain, both from human [29] and medTK*b* in this study. Thus, TK domains exist in a semi-constitutively active state, only blocked from activity by the flanking tails that act as a ‘plug’ (Figure 3A and B). In both medTK*b* and human TK, the formation of the essential salt bridge (*i*) between the invariant lysine in strand β3 (K68 in medTK*b*) and the conserved glutamate residue (E83) from helix ⍺C is prevented by the binding of the CRD tail into the ATP pocket, which displaces helix ⍺C into a tilted ‘open’ conformation. In medTK*b,* this displacement results in a distance of 4.92 ± 0.17 Å between the lateral groups of K68 and E83. Interatomic distances are also >4.9 Å in human TK. To date, no crystal structure of an uninhibited TK domain is available. This is due to the difficulty of producing viable recombinant samples in the absence of the flanking tails, which contribute significantly to kinase stability [30].

In medTK*b*, the sequence variations in the canonical DFG and AxK motifs that led to question the phosphotransfer activity of mammalian TKs are also present*,* here being ELG and MAK (Figure 3B and C). The ELG sequence of medTK*b* is also in the ‘in’ conformation (Figure 3B). The substitution of phenylalanine to leucine in this motif does not appear to have structural consequences and it still results in the formation of the hydrophobic R-spine (residues L87, L98, H157, L180). The second hydrophobic spine (C-spine) in canonical kinases is completed by the binding of ATP via the purine base moiety. Both in medTK*b* and human TK, the C-spine (residues V53, M66, I120, I165, V166, Y167, L224, L228) is completed by residue A328 from helix ⍺R2 of the CRD that packs into the ATP pocket and against the methionine residue in the MAK motif. A detailed description of this interaction and its possible implications for ATP binding is given in subsequent sections below. In brief, medTK*b* shares the same deviating features of human TK, retaining the pseudokinase character (Figure 3C).

A further feature of human TK that has complicated the exploration of its phosphotransfer activity is the speculated inhibition of its catalytic aspartate (D144; numbering as in PDB 4JNW [14]) by a tyrosine (Y187) from the *P*+1 loop to which it hydrogen bonds (Figure 3C and D) [5]. Both D144 and Y187 residues are embedded within a hydrogen bond relay network with residues R146, Q167 and R323. It is thought that the inhibition might be relieved by the phosphorylation of Y170 [5,20], similarly to kinases ERK2 [31] and IRK [32]. However, the presence of a tyrosine at this position is not necessarily inhibitory, as shown by the atypical, but active, kinase HASPIN that has a tyrosine in the *P*+1 loop that interacts with the catalytic aspartate (Figure 3D) but does not require phosphorylation for activation [33]. Thus, the inhibitory character of Y187 in human TK remains to be established. The tyrosine residue in the *P*+1 loop is conserved in medTK*a* (Figure 3C), where a predicted 3D model suggests that it also interacts with the catalytic aspartate (**Section S2**). However, the tyrosine residue is not present in medTK*b*, which contains instead an alanine residue (A202) in that position (Figure 3C and D). We conclude that tyrosine inhibition is not a general regulatory mechanism in TKs.

### Conservation in the N-terminal linker is limited to a YD kinase-binding motif

**Figure 4:**
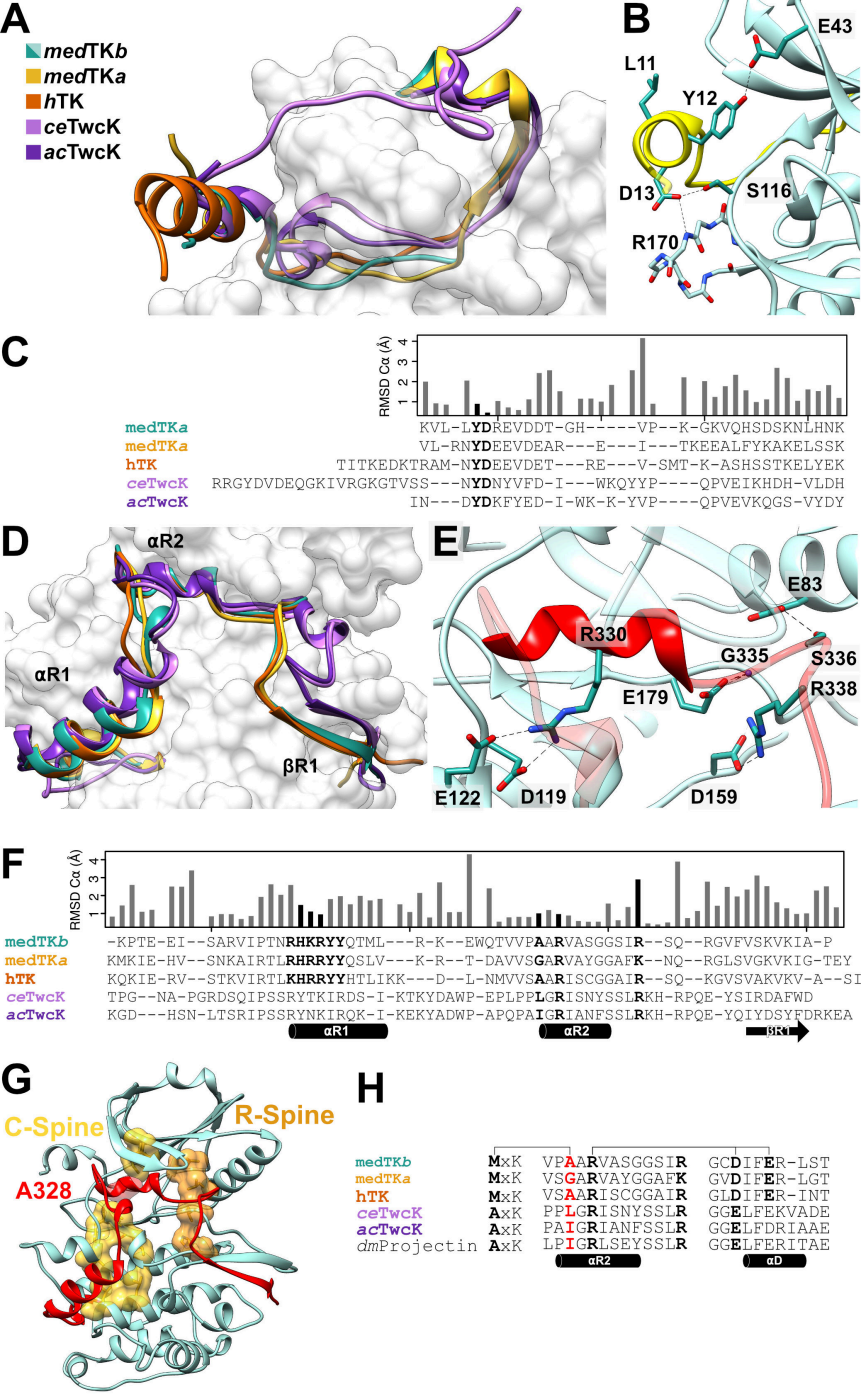
Comparison of N- and C-terminal tail extensions in titin(-like) kinases. **A.** N-terminal linker (NL) in available crystal structures of TK and TwcK homologues. Structures shown are excerpts from PDB codes: *ac*TwcK, 1KOB [8]; *ce*TwcK, 3UTO [9]; human TK, 6YGN [6]. A 3D-model of medTK*a* (yellow) predicted using ColabFold [39] is included for completeness; **B**. Conserved interactions of the YD motif (residues 12–13) in the NL of medTK*b*; **C**. Structure-based sequence alignment of NL segments accompanied of a histogram of per-residue RMSD values for C⍺ atoms across all kinases shown in the alignment (calculated using UCSD chimera; https://www.cgl.ucsf.edu/chimera). The conserved YD motif is highlighted; **D**. C-terminal regulatory domain (CRD) of TK and TwcK homologues. Colouring as in A.; **E**. Interactions supported by helix ⍺R2 in medTK*b*. In medTK*b*, an additional (non-conserved) interaction is that of residue S336 with the conserved glutamate (**E83**) from the catalytic ⍺-helix C; **F**. Structure-based sequence alignment of CRD tails. Details as in C. The conserved [R/K]H[R/K]RYY and R-7x-R motifs are highlighted as well as the residue in position -2 of the R-7x-R motif that packs against strand β3; **G**. Structure of medTK*b* with its pre-assembled catalytic (C-, yellow) and regulatory (R-, orange) spines shown in surface representation. The C-spine completing residue A328 from helix ⍺R2 of the CRD is indicated; **H**. Sequence alignment of helix ⍺R2 in TK and TK-like sarcomeric kinases showing The R-7x-R motif and the interactions it supports (connector lines). The C-spine completing residue in position -2 is shown in red. The correlated variation of this residue with the ATP-binding AxK motif in strand β3 is evident. In TwcK, the E/D residue in the hinge region does not form the annotated interaction.

In titin proteins, the TK domain is always flanked N-terminally by a linker sequence (NL), approx. 33 residues in length, that joins it to the preceding fibronectin domain in the chain. Even though its sequence composition is highly variable, the crystal structure of medTK*b* reveals that the NL tail is a structurally conserved feature of TK, consisting of a short N-terminal α-helix followed by an extended chain that wraps around the N-terminal kinase lobe (Figures 2A, 4A and B). Sequence conservation in the NL tail is limited to a short, strictly conserved YD motif, which is commonly preceded by an asparagine residue (73.8% of all TK sequences). Structurally, the motif binds against the inter-lobular hinge region of TK. In medTK*b*, the hydroxyl group of residue Y12 forms a hydrogen bond to the side chain of E43 from strand-β1 and D13 forms a hydrogen bond with residue S116 of the hinge-loop as well as the backbone amino group of R170 from the loop between strands β7 and β8 of the C-lobe (Figure 4B). The residues interacting with the YD-motif in medTK*b* (residues E43, S116 and R170) are conserved across the 459 TK sequences analysed in this study (99%, 99% and 96% sequence identity, respectively; including human TK), pointing to the high significance of this locus.

The YD motif is the primary responsible for the packing of the NL tail against the kinase domain, as shown by the mutation of the conserved D residue to valine in human TK that impaired the interaction [6]. Based on force-biased molecular dynamics simulations, the interactions held by the YD motif have been proposed to act as a force-barrier that protects the kinase domain from stretch-unfolding induced by pulling forces in the sarcomere [6]. The motif appears to confine stretch-unfolding to the NL tail, preventing the propagation of stretch along the protein chain and thereby protecting the kinase domain from mechanical damage. Interestingly, the single nucleotide variant (SNV) D24728V affecting the YD motif of human TK has been found in patients with dilated cardiomyopathy, supporting an important functional role of this motif in titin [6].

Notably, the YD motif and its interacting residues are also conserved in the TwcK homologue from invertebrates, showing the lowest RMSD values within the NL region when comparing available 3D structures of TK and TwcK representatives (Figure 4C). It is also invariably present in the sarcomeric homologues, TTN-1 kinase and projectin kinase (Supplementary Figure S3). This suggests an importance of this motif in titin-like kinases at large. Other than the YD motif, sequences of the NL tail show little conservation despite the structural similarity (Figure 4C).

### The C-terminal CRD extension plays multiple inhibitory and stabilizing roles

The CRD tail extension of human TK binds against the kinase domain blocking access to the active site [5,6,14] and critically contributes to kinase stability [30]. Structurally, the CRD tail folds into two helices (⍺-helix ⍺R1 and the 3_10_-helix ⍺R2) and a C-terminal β-strand (strand βR1) (nomenclature as in [5]) (Figure 4D). Helix ⍺R1 is thought to particularly contribute to TK’s fold stability [20,30]; this also being the case in titin-like kinases such as PK1 from *Drosophila* obscurin [35]. A feature of interest in helix ⍺R1 is the highly conserved motif [R/K]H[R/K]RYY (Figure 4F). The motif is partly solvent exposed on the surface of the helix, where it is freely accessible and makes a prime candidate for a binding interface. In human TK, this motif has been proposed to bind the autophagy receptor Nbr1 [20]. However, subsequent *in vitro* studies could not confirm the interaction [6,36]. Notably, the motif hosts an SNV (R32450W) that yields the sequence HWR [20]. The potential association of this SNV with hereditary myopathy with early respiratory failure (HMERF) is a matter of debate [37,38].

Helix ⍺R2 and strand βR1 are the primary inhibitory elements of the CRD tail. Helix ⍺R2 is a short 3_10_-helix that penetrates the ATP-binding pocket, while strand βR1 forms a two-stranded antiparallel β-sheet with the activation loop that blocks the binding of a potential peptide phosphorylation substrate. We observe that a conserved signature motif of this chain segment is RxxxxxxxR (here abbreviated as R-7x-R), where two arginine residues are separated by any 7 residues. The first R is in helix ⍺R2 and the second is in the loop between helix ⍺R2 and strand βR1 (Figure 4E and F). In medTK*b,* the first arginine (R330) forms a salt bridge with residues D119 and D122 in the kinase hinge-loop and the second arginine (R338) forms a salt bridge with the catalytic aspartate (D159) (Figure 4E). The interactions held by the first arginine are conserved across all TK and TwcK homologues of known 3D structure. However, whether the second arginine interacts or not with the catalytic aspartate depends on the presence or absence of the putatively inhibitory tyrosine rest in loop *P*+1. In the absence of the tyrosine rest, the arginine interacts with the catalytic aspartate as observed in medTK*b*, *ec*TwcK and *ac*TwcK (Figure 3D). In the presence of the tyrosine residue, the catalytic aspartate does not interact with the arginine from the CRD, but with the tyrosine rest, as seen in crystal structures of human TK (PDB: 1TKI [5], 4JNW [14], 6YGN [6]) (Figure 3D). Thus, the catalytic aspartate appears to be part of an interaction switch where either the tyrosine residue from the *P*+1 loop or the arginine from the CRD is engaged.

A further important feature of helix ⍺R2 is position -2 with respect to the R-7x-R motif. In the autoinhibited medTK*b*, human TK, *ec*TwcK and *ac*TwcK, this residue binds into the ATP binding pocket and against position 1 of the AxK motif, mimicking ATP binding and thereby completing the C-spine (Figure 4G). Because of the bulky methionine in TK, for this packing to occur efficiently, the residue from helix ⍺R2 must necessarily be small, commonly glycine or alanine. In TwcK kinases (as well as in the homologous TTN-1 kinases and projectin kinases), which are catalytically active and contain a canonical AxK motif, position -2 hosts a larger hydrophobic residue, commonly leucine or isoleucine (online supplementary material 2). Thus, the residue size at this CRD position strongly anti-correlates with the size of the first residue in the AxK motif of strand β3, the co-variation resulting from a volume compensation of these mutually packing groups (Figure 4H). In canonical kinases, the C-spine is completed by the nitrogenated base of the bound ATP substrate. Thus, upon ATP binding, the residue from the CRD must be substituted by the nitrogenated base moiety of the ATP. Our observations lead us to question whether the large ATP base could be well accommodated at this position in TK. Adding to previous speculations [14], the covariation of residues in strand β3 and helix αR2 in TKs supports the view that the A-to-M exchange in the AxK motif conflicts ATP binding in TK.

### Comparison of sequence-motifs within and across PaSiMap clusters

**Figure 5:**
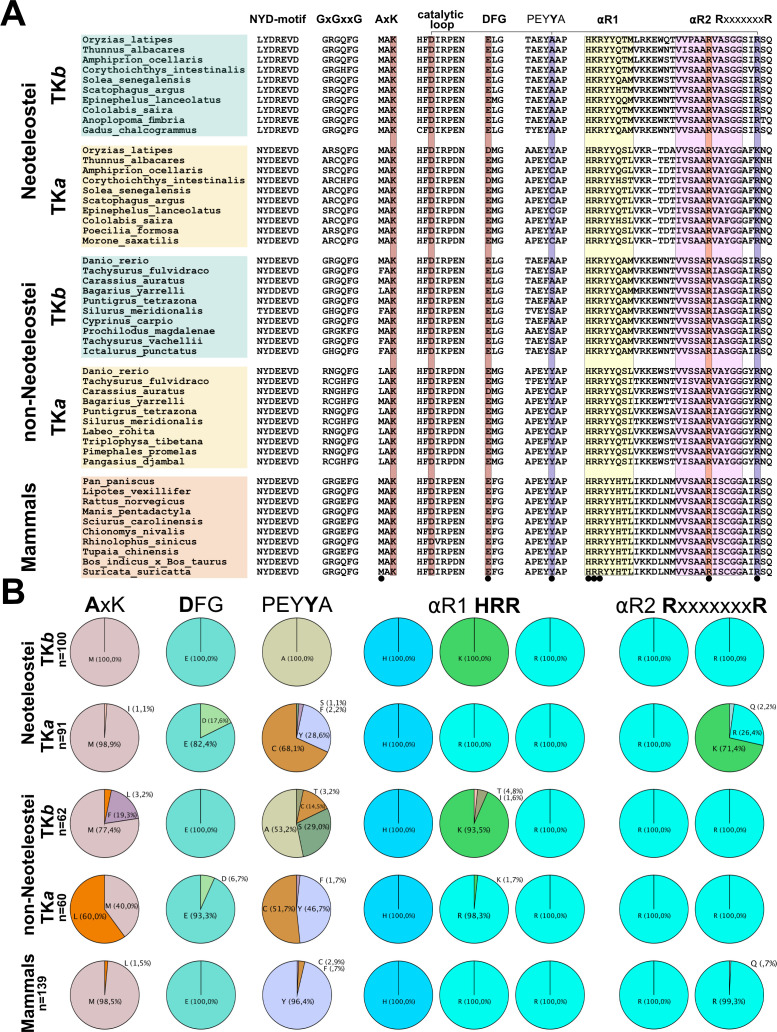
Comparison of conserved sequence motifs across PaSiMap TK clusters. Analysis of conservation in functional sequence motifs across the five clusters of TK sequences classed by PaSiMap (corresponding to TK isoforms *a* and *b* of neoteleostei, isoforms *a* and *b* of non-neoteleostei, and mammalian TK). **A**. Selected sequences for each group are shown. The sequence motifs are stated as headings in their canonical occurrence. Catalytically relevant residues are highlighted in brown; these are the lysine residue in the AxK motif, the aspartate in the catalytic loop and the D/E residue of the DFG-motif. The two alternative binders (tyrosine in *P*+1 loop or arginine from the CRD) of the catalytic aspartate are marked in blue and connected by a horizontal bar on top of the sequence. The CRD helix ⍺R1 is highlighted in yellow; **B**. Quantitation of conserved residues across the sequence groups is shown in percentages. The selected residue positions quantitated in the chart correspond to the residue in bold in the sequence above each chart. The same residues are also indicated with small black circles in the multiple sequence alignment in A.

Having explored sequence identifiers in crystal structures, we finally asked whether such features are representative of TK kinases at large or specific TK groups. To gain an insight into this question, we analysed sequence conservation in the identified PaSiMap clusters (Figure 5). We observed that the two deviating features of the kinase domain, i.e. the MxK and ExG motifs, are present throughout all TK groups. Specifically, all 452 TK sequences from fish and mammals contained a large hydrophobic residue in the first position of the MxK motif. TK from mammals (*n* = 139) and TK isoforms *a* and *b* from neoteleostei (*n* = 91 and *n* = 100, respectively) contain a methionine residue at this position in 98.5%, 98.9% and 100% of its representatives. TK isoforms *a* and *b* from the older (and, therefore, more diversified) non-neoteleostei group (*n* = 60 and *n* = 62, respectively) contain a methionine rest 40% and 77.4% of the times, respectively. Residues other than methionine across all groups were leucine (8.7%), isoleucine (0.6%) and phenylalanine (2.6%). It can be concluded that strand β3 of TK sequences invariably hosts a θxK motif, where θ represents a bulky hydrophobic residue. Accordingly, position -2 in helix ⍺R2 of the CRD is consistently a small residue, most commonly an alanine across all groups (76.8% alanine, 15.2% serine/threonine, 4.8% glycine, 1.5% valine, only rarely 0.4% being a larger glutamate or glutamine residue). This sequence covariation is a notable feature of TK.

In contrast, the ExG motif presents a diversification that somewhat correlates with TK isoform. Mammalian TK and all isoforms *b* (from both neoteleostei and non-neoteleostei) contain a glutamate residue (E) 100% of the time. TK isoform *a* largely contains a glutamate (E), but a number of representatives (including medTK*a*; Supp Section S2) contain a canonical aspartate (D); namely, 17.6% and 6.7% occurrences in neoteleostei and non-neoteleostei, respectively (amounting to 20 out of 452 TK sequences in this study). Whether these TK*a* variants might deliver phosphotransfer catalysis or might have lost this capability through another mechanism is yet to be investigated. In that respect, it must be noted that sequence variations in the ATP-binding GxGxxG loop also correlate with TK isoform. Mammalian TK and fish isoform *b* (from both neoteleostei and non-neoteleostei) have a conventional GxGxxG motif, while TK*a* variants from both fish groups have diversified loops. TK*a* from non-neoteleostei exhibit a RxGxxG motif while TK*a* from neoteleostei have an Ax[C/S]xxG sequence. In contrast to these general features, rather modest conservation is found in the potentially inhibitory tyrosine from the *P*+1 loop. This residue is highly conserved only in mammals (96.4%), being modestly present in TK*a* isoforms from fish (28,6% in neoteleostei and 46.7% in non-neoteleostei) and totally absent from TK*b* isoforms. Thus, any functional significance of this residue might be primarily specific to mammalian TK.

Remarkably, a very high conservation of specific features in the NL and CRD flanking tails is also observed. In the NL segment, the [N/L]YD motif is strictly present in all TK sequences here studied, irrespective of animal group. The CRD tail mimics both ATP and peptide binding, completing the C-spine and maintaining the inhibited kinase in a primed catalytic conformation. In this tail, the almost strict conservation of the [K/R]H[R/K]RYY motif in helix ⍺R1 of all TK sequences is also striking, especially as the two central positively charged residues (99% K/R and 100% R, underlined) are exposed to solvent and facing away from the kinase domain. The conservation of the R-7x-R motif in helix αR2 is also striking and can be explained by the role that this motif plays in the packing of the tail against the kinase domain.

Taken together, the motifs here described constitute the determinants of TK and jointly define its conserved pseudokinase character.

## Discussion

Sarcomeric kinases are a group of kinases and pseudokinases thought to play mechanosensory roles in muscle. The best studied representatives of this group are the close homologues human TK and TwcK from *C. elegans*. TwcK is an active kinase that undergoes autophosphorylation [40] and has demonstrated activity on model substrates [9,41]. The ablation of TwcK’s phosphotransfer activity through the inactivating point mutation K-to-A has a phenotypic outcome in which *C. elegans* worms swim at an accelerated pace yet display reduced overall fitness [42]. In contrast, human TK is a pseudokinase and while its scaffolding functions are well established [6,14,20], its phosphotransfer activity is thought to be abolished or highly reduced [14]. Accordingly, no validated substrate has been established for TK to date [30]. Here, we set out to examine sequence features in TK across fish and mammals that cover long evolutionary distances, aiming to reveal if the pseudokinase character of TK is conserved through evolution.

Our study reveals that, despite sequence variations, TK domains are strongly conserved structurally including their flanking tails, and that their pseudokinase features are preserved through evolution. In particular, the θxK motif (where θ is a bulky, hydrophobic residue) and the EFG motif, both thought to hinder phosphotransfer activity, are largely conserved throughout TK sequences. We rationalize the conservation of the θ residue in the first motif as a covariation with the presence of a small residue in the CRD sequence that penetrates the ATP pocket and packs against it, adding to the notion that TK cannot bind ATP in a canonical fashion. Using a previously reported multiple sequence alignment of human kinases [43], we identified that RIPK1 [44], NEK8 [45] and Eif2ak4 [46] kinases contain isoleucines in position θ and LMTK2 [47] contains leucine, yet all are active kinases. The ATR-kinase contains a methionine as θ residue and is also proposed to be active [48]. On the other hand, RYK kinase contains a phenylalanine residue in this position and is known to be catalytically inactive [49], not binding nucleotides or cations detectably [50]. Experimental validation of the impact of the methionine residue on TK’s phosphotransfer activity is awaited.

In contrast, the reason for the strict conservation of the glutamate residue in the EFG motif is unclear. Although the glutamate side chain binds to the backbone of a conserved glycine residue in the CRD tail (Supplementary Figure S4), this interaction does not explain conservation. In *ce*TwcK, the canonical aspartate interacts with water molecules in the CRD-bound state (Supplementary Figure S4). As evolutionarily only those protein residues with functional implications need to be retained, lacking the constraint of performing phosphotransfer allows pseudokinases to diverge in catalytically critical residues [50,51]. If the physiological functionality of TK did not require it to display phosphotransfer activity and given the absence of structural constraints, a variety of inactivating residues might occur in lieu of E/D at this position. The high conservation of the glutamate residue in this motif and its chemical resemblance to the canonical aspartate lead us to speculate that this might be a compensatory residue variation correlated to the θxK motif and possibly directed to enable certain phosphotransfer activity. In this regard, it is interesting to note that our analysis has identified a set of 20 TK sequences of isoform *a* from fish (including that of the model organism medaka) that contain a canonical DFG motif. All 20 kinases have the θxK motif (16 sequences contain M, 4 sequences L). A future characterization of these TK variants will be highly informative to further elucidate the phosphotransfer properties of TK.

Beyond the kinase domain, the NL and CRD extensions of TKs show remarkable structural similarity even though their sequence conservation is modest. Conservation is primarily limited to four motifs: (*i*) the YD-motif of the NL; (*ii*) a [R/K]H[R/K]RYY motif in helix ⍺R1 of the CRD; (*iii*) a R-7x-R motif in the CRD helix ⍺R2; and (*iv*) a small residue in position -2 of the R-7x-R motif. With the exception of motif [R/K]H[R/K]RYY (*ii*), whose function is unknown, the role of the conserved motifs (*i, iii, iv*) is to mediate the packing of the tails against the kinase domain. The YD-motif of the NL is the primary anchor of the NL onto the kinase domain, its mutation disrupting the interaction [6] . The R-7x-R motif and its -2 position anchor helix ⍺R2 from the CRD onto the kinase and into the ATP binding pocket. In TK, the hydrophobic θ residue of the θxK motif and the small residue in position -2 jointly complete the C-spine. In the active TwcK from invertebrates containing a canonical AxK motif, position -2 houses a larger hydrophobic residue (commonly Leu or Ile) that completes the C-spine. Notably, the conserved motifs in NL and CRD extensions as well as a residue composition of position -2 that segregates with phosphotransfer activity are also observed in the close homologues TTN-1 and projectin active kinases (online supplementary material 2). We conclude that there are structural constraints on the residue in position -2 and that its divergence is part of the pseudokinase sequence signature of TK. Moreover, the conservation in NL and CRD tails suggests that the mechanosensory mechanism of TK through force-induced tail unraveling is conserved across vertebrates.

Finally, we observe a distinct segregation of TK isoforms in fish, evidenced by sequence clustering (Figure 1A and C). The segregation of TK sequences resembles that of full-length titin in a selected set chosen for analysis (Supplementary Figure S1). This leads us to propose that TK domains can be utilized as a proxy in the classification of titin proteins from fish, facilitating the task for such a large protein. Adopting the titin gene nomenclature from zebrafish, we propose a classification of so far unannotated titin genes into *a* and *b* isoforms (*ttna* and *ttnb*) that can assist future studies. A curated gene annotation is provided here as supplementary material (Supplementary Table S1). Finally, the molecular analysis presented here furthers our understanding of titin across mammalian, zebrafish and medaka model systems, advancing our interpretation of physiological data derived from their study.

## Methods

### Cloning

DNA coding for the TK domain of ttnb from *Oryzias latipes*, medTK*b*, (residues 27887–28241; NCBI XP_023806503) was synthesized commercially (Biocat, DE). The gene was cloned into the vector pETtrx1a (J. Bogomolovas) using NcoI and KpnI restriction sites, as described previously for human TK [30]. This vector fuses a His_6_-tagged thioredoxin protein and a tobacco etch virus (TEV) protease cleavage site N-terminally to the gene insert of interest. The construct was confirmed by sequencing (Eurofins, DE).

To ease structural annotation and facilitate comparison with previously reported crystal structures of human TK, residue M27887 in entry XP_023806503 is set as residue 1 in this study.

### Protein production

MedTK*b* was expressed recombinantly in *E. coli* SoluBL21 (Amsbio, U.K.) in TB medium supplemented with 50 μg/ml kanamycin, as described for human TK [30]. Cultures were grown at 37°C to an OD_600_ ≥ 1. Upon cooling to 18°C, expression was induced with 0.5 mM IPTG and growth continued for a further 18 h. Cells were harvested by centrifugation. The pellet was resuspended in 20 mM HEPES pH 8.0, 250 mM NaCl, 5 mM imidazole, 0.2% Triton-X-100, 1 mM dithiothreitol (DTT), supplemented with cOmplete^TM^ ULTRA EDTA-free protease inhibitors (Roche, CH). Cell lysis was done by sonication and the lysate subsequently clarified by centrifugation. The supernatant was applied to a Ni^2+^ HisTrap HP column (Cytiva, SE) equilibrated in the buffer described above and proteins eluted with buffer supplemented with 300 mM imidazole without detergent. Tag removal was by incubation with TEV protease in 25 mM Tris pH 8.0, 50 mM NaCl, 1 mM DTT, overnight at 4°C. Further purification used ion exchange chromatography on a HiTrap SP column (Cytiva, SE) and gel filtration on a Sephadex S200 16/60 column (GE Healthcare, DE) in 25 mM Tris-HCl pH 8.0, 50 mM NaCl, 1 mM DTT. Samples were stored at 4°C until further use.

### X-ray crystallography

The medTK*b* sample was concentrated to 11.6 mg/ml and used in crystallization trials that used the sitting drop vapour diffusion method in ARI Intelliplates (Art Robbins Instruments, U.S.A.) incubated at 18°C. Drop composition was a mixture of 100 nl protein : 100 nl reservoir solutions. Crystallizability was assayed using the commercial screens: Morpheus (MD1-46), Structure Screen 1&2 Eco (MD1-01-Eco, MD1-02-Eco), JCSG plus Eco (MD1-37-ECO) and Proplex (MD1-38-ECO) from Molecular Dimensions; Wizard 1&2 (EB-W12-T, EB-W34-T) from Emerald Biosystems/Rigaku; and JBScreen Basic (CS-125) from Jena Bioscience. Crystals of appropriate diffraction quality grew from 20% [w/v] PEG 8000, 100 mM HEPES pH 7.5. For X-ray data collection, crystals were flash-frozen in liquid nitrogen using mother liquor supplemented with 30% [v/v] ethylene glycol as cryo-protectant. X-ray diffraction data were collected at beamline P14 (EMBL c/o DESY, Hamburg, DE) under cryo-conditions (100 K). For data processing, the XDS/XSCALE suite was used [52]. Phasing was done by molecular replacement in PHASER [53] using human TK as search model (PDB code 4JNW [14]). An initial atomic model was built automatically using Arp/wArp [54] and further manual building was performed in COOT [55]. Model refinement was carried out in Phenix.refine [56] applying TLS refinement and non-crystallographic symmetry (NCS), with each chain being a TLS and NCS group. X-ray data statistics and model parameters are given in Table 1. The first eleven residues of chain A and the first nine of chain B (of which the first two residues originate from the vector) are not visible in electron density maps. As a result, the first residues contained in the crystal structures are L10 and K8 (residue L27896 and K27894 in NCBI XP_023806503) for chains A and B, respectively.

### Sequence comparison

The sequence of human TK, including its NL and CRD segments, (UniportKB: Q8WZ42, residues 32150–32491) was used as query in a blast search of related sequences (https://blast.ncbi.nlm.nih.gov/Blast.cgi) that yielded 5034 entries. The sequences were pruned to include proteins from vertebrates only. In addition, entries labelled as myosin light chain kinase or annotated as LOW_QUALITY were excluded. Finally, the sequences were made unique to exclude redundancy deriving from different titin isoforms with sequence variations in regions other than the kinase domain. This left 738 sequences including the clades of reptiles, fish, amphibians and mammals; 459 of those were sequences from fish and mammals, of which 320 sequences belonged to fish.

The 320 sequences from fish were clustered using PaSiMap [24] implemented in JalView [25]. Cluster plots were generated with RStudio (https://posit.co/download/rstudio-desktop). As this revealed that fish sequences segregated into different clusters, a next analysis clustered all 459 sequences of fish and mammals to explore if mammalian TK is similar to one of the fish groups or forms its own cluster.

Finally, to quantitate the natural residue variability at functional loci of the kinase fold, the 459 sequences were aligned with ClustalO ( https://www.ebi.ac.uk/jdispatcher/msa/clustalo?stype=protein ). Partial sequences that did not cover the full kinase region were removed (leaving 452 sequences) and then residue type per position within the multiple sequence alignment quantified and illustrated using pie charts.

## Supplementary material

online supplementary material 1.

online supplementary material 2.

## Data Availability

Structure co-ordinates and experimental diffraction data have been deposited with the PDB (entry 9QJ2) [57]. X-ray diffraction images have been deposited with Zenodo (http://doi.org/10.5281/zenodo.14922781) [58].

## Open Access

This article has been published open access under our Subscribe to Open programme, made possible through the support of our subscribing institutions, learn more here:

## Abbreviations

CRD: C-terminal regulatory domain
FRET: fluorescence resonance energy transfer
NL: N-terminal linker
TK: titin kinase
