# Supplementary material for: Molecular identifiers of the evolutionarily conserved titin pseudokinase
Source: Biochem J. 2026 Jan 7;483(1):BCJ20253442. doi: 10.1042/BCJ20253442 (PMC12862963; doi:10.1042/BCJ20253442)
Supplement: online supplementary material 2. [file bcj-483-01-BCJ20253442-s002.pdf]

Supplementary Materials

Molecular identifiers of the evolutionarily conserved titin pseudokinase

Dorendorf et al., 2025

SuppMat_TableS1-GeneNames.xlsx

List of protein entries used in the sequence comparison in this study. Entries are grouped according to their PaSiMap cluster classification, where groups correspond to mammals, neoteleostei TK isoform a (TK*a*), non-neoteleostei TK*a*, neoteleostei TK isoform b (TK*b*) and non-neoteleostei TK*b*. For each column the NCBI database access code to the gene is given, followed by the residue range corresponding to the TK sequence analyzed, the gene name and the source organism.

SuppMat_S2 (3DPaSiMap_Fish_TK.html): Interactive PaSiMap vector map revealing similarity clusters of TK sequences from fish. Shown are dimensions 2, 3 and 4 of the PaSiMap cluster map of 322 TK sequences from fish. Circles and empty diamonds indicate sequences from neoteleostei and non-neoteleostei, respectively. Yellow corresponds to TK isoform *a* and teal to TK isoform *b*, as named in this manuscript. A black cross indicates the origin of coordinates. (Plot created in R-Studio; http://www.rstudio.com/). Clicking a data point reveals the identity of the protein sequence which it represents.

SuppMat_S3 (3DPaSiMap_Fish_Mammals_TK.html): Interactive PaSiMap vector map revealing TK sequence clusters from fish and mammals. Shown are dimensions 2, 3 and 4 of the PaSiMap vector map of 459 TK sequences from fish and mammals. Mammalian TK are brown squares, circles and diamonds indicate sequences from neoteleostei and non-neoteleostei, respectively. Yellow corresponds to TK isoform *a* and teal to TK isoform *b*, as named in this manuscript. A black cross indicates the origin of coordinates. (Plot created in R-Studio; http://www.rstudio.com/). Clicking a data point reveals the identity of the protein sequence which it represents.

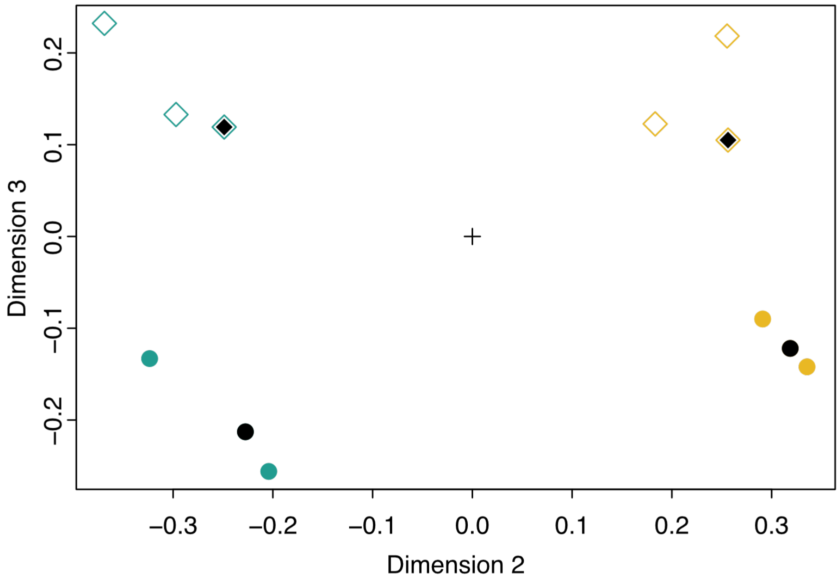

Figure S1: PaSiMap vector map of full-length titin protein sequences from selected fish representatives.

PaSiMap analysis of full-length titin isoforms from the neoteleostei *Amphiprion ocellaris, Oryzias latipes* and *Thunnus albacares*; and non-neoteleostei *Danio rerio*, *Tachysurus fulvidraco* and *Carassius auratus*. Fish groupings are indicated by rhombic (non-neoteleostei) and circular (neoteleostei) data points. The model organisms representative of each group, Zebrafish (*Danio rerio*; ◆) and medaka (*Oryzias latipes*; ●) are indicated with black symbols. Yellow and teal colours indicate isoforms *a* and *b* as deduced from the clustering of titin kinase sequences in Fig 1A. The result shows that the clustering of full-length titin sequences reproduces the clustering of titin kinase sequences, so that the TK domain is a valid surrogate for the classification of full-length titin protein isoforms. Based on this analysis, we conclude that entries corresponding to titin isoform a are: *Amphiprion ocellaris* (XP_054871008.1), *Oryzias latipes* (XP_023806459.1), *Thunnus albacares* (XP_044221301.1), *Danio rerio* (ABG48500.1), *Tachysurus fulvidraco* (XP_047670400.1), *Carassius auratus* (XP_026074665.1); isoforms b are *Amphiprion ocellaris* (XP_054871216.1), *Oryzias latipes* (XP_023806503.1), *Thunnus albacares* (XP_044222812.1), *Danio rerio* (ABG48499.1), *Tachysurus fulvidraco* (XP_047670412.1) and *Carassius auratus* (XP_026127101.1).

Section S2: Medaka isoform TK*a* contains a canonical DMG motif

To complete the analysis of TK kinases in medaka, we predicted the 3D-structure of the medTK*a* isoform using AlphaFold (**Figure S2A**) (Mirdita et al., 2022) and compare it to the experimental structures of human TK and medTK*b* (**Figure S2B**). The predicted model is in close agreement with the crystal structures of medTK*b* and human TK ($RMSD_{C\alpha}$=1.24 Å across all 344 aligned C_α-atoms to medTK*b* and $RMSD_{C\alpha}$=0.89 Å across all 344 aligned C_α-atoms to human TK [PDB: 6YGN], calculated using UCSD Chimera; https://www.cgl.ucsf.edu/chimera). The model of medTK*a* displays the same features as described in this work for medTK*b* and human TK. The hydrophobic spines are pre-formed, where V46, M59, I113, IVY158-160, M217 and L221 form the C-spine (M217 is equivalent to L224 in medTK*b*) and the residue in position -2 of helix αR2 from the CRD-tail is a glycine (G320). The R-spine is composed of L80, L91, H150 and M173, where M173 is part of the canonical DFG-motif that here is DMG (ELG in medTK*b*). The DMG motif of medTK*a* contains the canonical aspartate and is also found in human CDK19 and CDK8, the latter being an active kinase (Fryer et al., 2004). As human TK, medTK*a* has a tyrosine residue in the P+1 loop that is predicted to hydrogen bond to the catalytic aspartate (**Figure S2D**). The glycine-rich loop in medTK*a* contains a ARSQFG sequence that differs from the canonical GxGxxG motif, but that is not at odds with ATP-binding as this loop can accept a variety of residues (Matsunaga et al., 2024). In brief, medTK*a* shares the MxK atypical motif with human TK and medTK*b,* as well as sharing the presence of a potentially inhibitory tyrosine residue in loop P+1 with human TK. However, contrary to human TK and medTK*b*, it lacks the DtoE exchange in the DFG motif, having a canonical aspartate in this position. A future investigation of whether medTK*a* presents increased levels of phosphotransfer activity compared to medTK*b* and human TK is required to establish the functional significance of this observation.

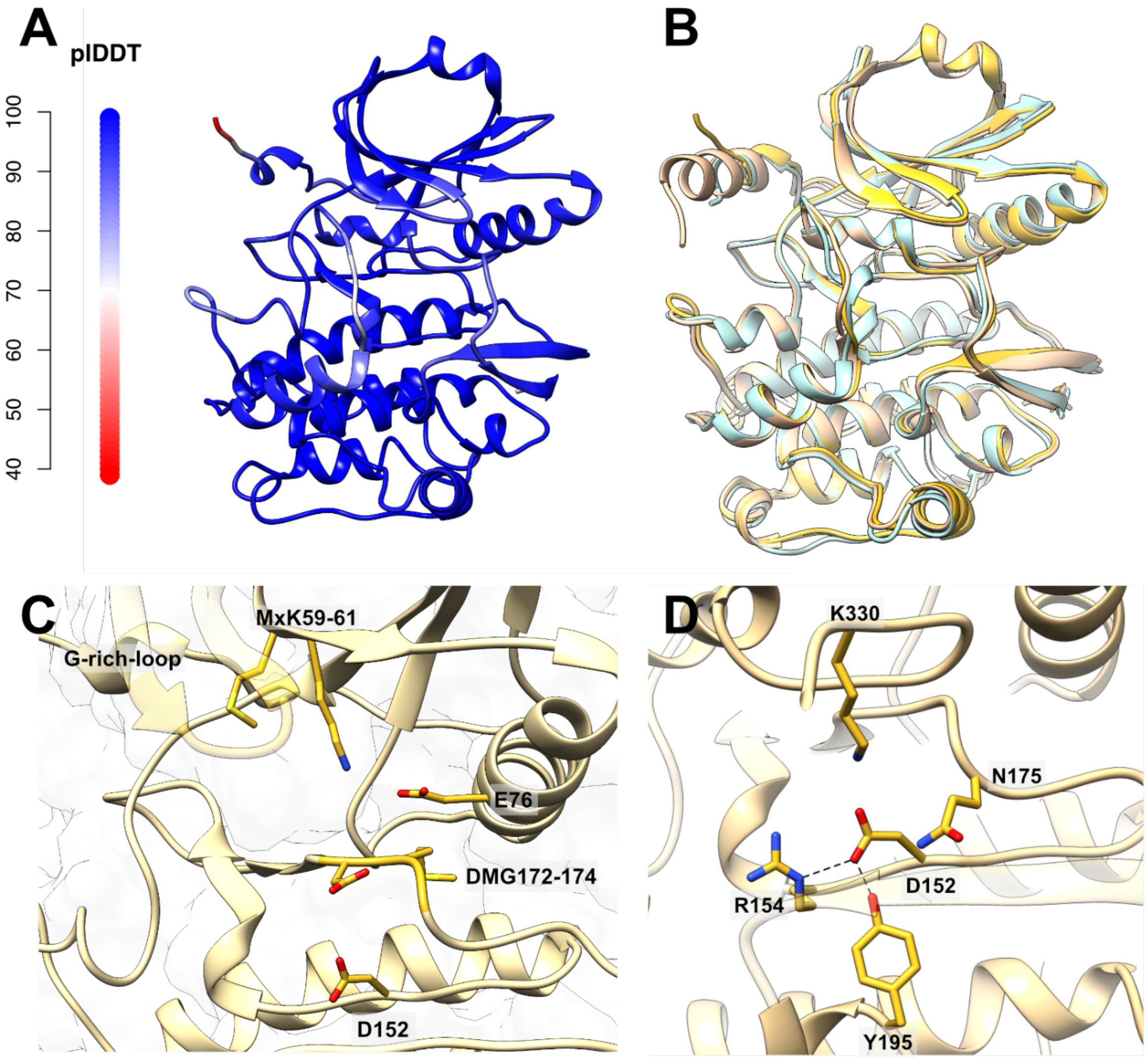

Figure S2: AlphaFold prediction of the 3D-model of *med*TK*a*.

A. 3D-model of *med*TK*a* predicted using AlphaFold2 (https://colab.research.google.com/github/sokrypton/ColabFold/blob/main/AlphaFold2.ipynb; Mirdita et al., 2022) (NCBI XP_023806459.1; titin isoform X10; residues 29360-29710). The structure is coloured according to prediction confidence, where the colour gradient corresponds to the pLDDT score. A pLDDT below 70 indicates unreliable modelling; **B.** Superimposition of the model predicted for *med*TK*a* (yellow) with the crystal structures of *med*TK*b* (light blue) and human TK (PDB: 6YGN; beige). $RMSD_{C\alpha}$=1.24 Å across all 344 aligned C_{α}-atoms to medTK*b* and $RMSD_{C\alpha}$=0.89 Å across all 344 aligned C_{α}-atoms to human TK, calculated using UCSD Chimera; https://www.cgl.ucsf.edu/chimera); **C.** Functional groups of the predicted medTK*a*. The CRD tail is removed for visual clarity; **D** Interactions sustained by the catalytic aspartate in the predicted 3D-model *med*TK*a*. Hydrogen bonds are shown as dotted lines.

Figure S3: Conservation of NL and CRD motifs in titin-like kinases from invertebrates

Sequence alignment of a set of sarcomeric, titin-like kinases from invertebrates: namely, twitchin kinase and TTN-1 kinase from nematodes and projectin from insects. In the NL, the [N/D]YD motif is a distinguished conserved feature. In the CRD, the R-7x-R motif and the bulky hydrophobic residue in position -2 are conserved, although in TTN-1 kinase the motif adopts the form R-8x-R. It must be noted that no crystal structure of a TTN-1 kinase is currently available that permits validating the structural equivalence of R-7x-R and R-8x-R motifs. Because of the remarkable resemble of the latter, we highlight here the motif in TTN-1 kinase in wait of its future investigation.

NL

```
medTKb                                                          LYDREVDDTGHVPKGKVQHSDSKNLHNK--YA

twitchin
Caenorhabditis elegans:NP_502274.2:6233-6584                    NYDNYVFDIWKQYYPQPVEIKHDHVLDH--YD
Auanema freiburgensis:AZI15602.1:3170-3521                      NYDNYVIDVWKQYYPQNIEIKHESVLDQ--YD
Necator americanus:XP_064052728.1:5873-6223                     NYDAYVIDVWKQYYPQPVEIKHDSVLDH--YD
Oesophagostomum dentatum:KHJ96255.1:758-1108                    NYDPYVIDIWKQYYPEPVEIKHDSVLDH--YD
Loa loa:XP_020306561.1:5925-6276                                NYDAYVIDVWKQYYPQSVEPKRESVYDY--YD
Dracunculus medinensis:VDN57410.1:5753-6102                     NYDSYVFDVWKEYYPQPVETKRESIYDY--YD
Enterobius vermicularis:VDD90847.1:5325-5680                    NYDSYVIDVWKQYYPQPVEPKKASVYDY--YD
Aplysia californica:1KOB:25-374                                 DYDKFYEDIWKKYVPQPVEVKQGSVYDY--YD
Aphelenchoides besseyi:KAI6211826.1:6057-6405                   NYDHYVFDIWKQEAPLPVDIKKHSIYHD--YD
Dreissena polymorpha:KAH3877134.1:259-608                       DYDKFYYDLWKKYVPQPVEIKQDSVYDY--YD

Projectin
Drosophila melanogaster:NP_001284716.1:7602-7980             PVKDYDSYVFDIYSKFVPQPVEISQQSVYDR--YD
Drosophila simulans:XP_016037335.1:7650-8028                 PVKDYDSYVFDIYSKFVPQPVEISQQSVYDR--YD
Drosophila yakuba:XP_015046889.1:7834-8212                   PVKDYDSYVFDIYSKFVPQPVEISQQSVYDR--YD
Bactrocera dorsalis:XP_049315862.1:7941-8319                 PIKDYDSYVFDIYSKFVPQPVEISHDGVYDK--YD
Episyrphus balteatus:XP_055858169.1:8026-8395                PIKDYDSYVFDIYSKYVPQPVEISHDSVYDR--YE
Eupeodes corollae:XP_055922644.1:7986-8355                   PIKDYDSYVFDIYSKYVPQPVEISHDSVYDR--YD
Chrysoperla carnea:XP_044740884.1:8054-8426                  PIHDYDQYVFDIYSKYVPQPVEISNRSVYDD--YD
Vespa mandarinia:XP_035738991.1:8171-8543                    KVRDYDQFVFDIYSKYVPQPVEIKHTSVYDK--YD
Vespa velutina:XP_047354416.1:8162-8534                      KVRDYDQFVFDIYSKYVPQPVEIKHTSVYDK--YD
Musca domestica:XP_058982941.1:7637-8015                     IVKDYDSFVFDIYTRYVPQPVEISRESVYDK--YE

TTN-1
Caenorhabditis elegans:NP_001367912.1:12100-12501               DYERVAKDSEPSE-YKTIDIHRLPNDLQAKYI
Auanema sp. JU1783:CAI4227244.1:171-573                         NYDKLDTKVEPVD-YKTVDVHKLPNDLHSKYI
Ancylostoma ceylanicum:EYC10057.1:215-616                       DYDRLDSKVDPSD-HKLIDIHHLPNDLQAKYI
Teladorsagia circumcincta:PIO76038.1:207-607                    NYDRLDAKVDLTK-HKPIDINRLPNDLQAKYI
Necator americanus:XP_064057964.1:201-602                       DYDKLDAKVDHSD-HKIIDINHLPNDLQAKYI
Cylicocyclus nassatus:CAJ0590415.1:240-638                      DYDRLDAKVAHDDKNVVIDINHLPNDLQAKYI
Diploscapter pachys:PAV80611.1:210-593                          DYDKLKTD--VSE-YKTVDVHRLPKDLQAKYT
Loa loa:XP_020305443.1:205-591                                  NYDELFTDVKPLE-YRDIDMLKLSTDFESKYI
```

CRD

```
                                                      αR1                    αR2             βR1
medTKb                                       T----KPTEEISARVIPTNRHKRYYQTMLRKEWQTVVPAARVASGGSIRSQRGVFVSKVKIAP
                                                   αR1                     αR2      αR2'     βR1
twitchin
Caenorhabditis elegans:NP_502274.2:6233-6584      LTPGNAPGRDSQIPSSRYTKIRDSIKTKYDAWPEPLPPLGRISNYSSLRKHRPQEYSIRDAFWDRSEA
Auanema freiburgensis:AZI15602.1:3170-3521        LSSGYTASRDGQIPSSRYHGIRDSIRTKYDAWPEPNPAIGRISNYSSLRQHRPAEYKIHDAFFDRTEA
Necator americanus:XP_064052728.1:5873-6223       LSGSDTGSDN-VIPSSRYTNIRDRIKLKYDAWPEPMPPLGRISNFSSLRKHRPAEYHMHDAFFDRSEA
Oesophagostomum dentatum:KHJ96255.1:758-1108      LASGEVGGGD-VIPSSRYTDFRDKIRMKYDAWPEPVPPLGRISNFSSLRKHRPAEYHIHDAFFDRSEA
Loa loa:XP_020306561.1:5925-6276                  LSGVANNVPSQQIPNERYHSIRDSVRQRYDAWPEPNPPLGRISNYSSLKKHRPTEYHIHDTWFSREEG
Dracunculus medinensis:VDN57410.1:5753-6102       LNDIP--KKSEQIPSEKYYSVRDSVKQRYSAWPEPNPSLGRISNFSSLKIHRPDEYRIHDAWFNREEG
Enterobius vermicularis:VDD90847.1:5325-5680      LNNAPGSSASERIPSERYHSIRDSIRHKYDAWPEPNPPLGRISNYSSLRKHRPAEYHIHDAWFNREEG
Aplysia californica:1KOB:25-374                   LKGDHS-NLTSRIPSSRYNKIRQKIKEKYADWPAPQPAIGRIANFSSLRKHRPQEYQIYDSYFDRKEA
Aphelenchoides besseyi:KAI6211826.1:6057-6405     LRGHQ--GRDEQIPSSRYHQVRDSVRERYDSWPDPNPPIGRVANFSSLRTQRPSEYSIFETLFERSCA
Dreissena polymorpha:KAH3877134.1:259-608         LKGDHS-DRTHRIPASRYDKIRARIQARYADWPLPMPAIGRLANYSSLRKHNPKEYCIYDAYFDRREA

Projectin
Drosophila melanogaster:NP_001284716.1:7602-7980  L-TGDHSAMKQEINRDRYLAYREKLRRKYEDFERFLLPIGRLSEYSSLRKLLMEKYKIHDAVFDRRQAA
Drosophila simulans:XP_016037335.1:7650-8028      L-TGDHSAMKQEINRDRYLAYREKLRRKYEDFERYLLPIGRLSEYSSLRKLLMEKYKIHDAVFDRRQAA
Drosophila yakuba:XP_015046889.1:7834-8212        L-TGEHCAMKQIINRDRYLAYREKLRRKYEDFERYLLPIGRLSEYSSLRKLLMEKYKIHDAVFDRRQAA
Bactrocera dorsalis:XP_049315862.1:7941-8319      L-SGNYGDRSEQISRDRYLAFRDKLRKKYEDFNKYLLPIGRLSEYSSLRKLLMEKYKMHETSFDRRQAA
Episyrphus balteatus:XP_055858169.1:8026-8395     L-TGDHSDRTQKIAREKYLAFRDKLRKKYEDFNKYLLPIGRLSEYSSLRKLLMEKYKIHETSIDRRQAA
Eupeodes corollae:XP_055922644.1:7986-8355        L-MGDHSDRTQKIAREKYLAFRDKLRKKYEDFNKYLLPIGRLSEYSSLRKLLMEKYKIHETSIDRRQAA
Chrysoperla carnea:XP_044740884.1:8054-8426       L-TGDHKDKTKPIAASRYTNFRDKIRAKYDHWENYVLPIGRLSEYSSLRKLLIEKYKMHETSFDRRQAA
Vespa mandarinia:XP_035738991.1:8171-8543         L-TGDHSNRTNVISSSRYLSFRDKLRAKYENWDKYVLPIGRLAEYSSLRKLLIEKYKIYDSSFDRRQAA
Vespa velutina:XP_047354416.1:8162-8534           L-TGDHSNRTNVISSSRYLSFRDKLRAKYENWDKYVLPIGRLAEYSSLRKLLIEKYKIYDSSFDRRQAA
Musca domestica:XP_058982941.1:7637-8015          L-TGDHADRNKQIQRSRYLAFRDKLRMRNQDAGNYVIPIGRISEYSSLRKLLIDKYKICDTTFDRRQAA

TTN-1
Caenorhabditis elegans:NP_001367912.1:12100-12467 ITGP   MQPKLDKSG-VPARQKRNFLSLKRWSDDLLPIGRLAKRGAIFRRLTMDGVFERNIAFDTD-AA
Auanema sp. JU1783:CAI4227244.1:171-539           IAGP   MTPPADGGN-VPARQKRNFLSLKRWSDDLLPIGRMAKRGAIFRRLTMDGVFERNVSFDTDSSA
Ancylostoma ceylanicum:EYC10057.1:215-583         ITGP   TQPSPTGRGPLPTRQKKNFMSLKRWSDDLLPIGRLAKRGAIFRRLTMDGVFERNISFEAE-CA
Teladorsagia circumcincta:PIO76038.1:207-574      ITGP   TQP-AEGSGPLPTRQKKNFMSLQRWSDDLLPIGRLANRGAIFRRLTMDGVFERNITFETE-YP
Necator americanus:XP_064057964.1:201-579         ITGP   TQPFPAGRGPLPTRQKKNFLALKRWSDDLLPIGRLAKRGAIFRRLTMDGVFERNISFETE-CA
Cylicocyclus nassatus:CAJ0590415.1:240-605        MTGP   MQQSPSDRGPLPPRQKKNFMALKRWSDDLLPIGRLAKRGAIFRRLTMDGVFERNITFEAE-CA
Diploscapter pachys:PAV80611.1:210-560            IT--   TQPKADRGA-LPTRQKKNFLSLKRWSDDLLPIGRIAKRGAIFRRLTMDGVFERNIKFDTD-AA
Loa loa:XP_020305443.1:205-557                    ITT-   -TKLKAKRGKMLLAKKKDYIVRKNFSDVLIPIGKLAKTGAIFRRRSMDGTFERNIKFEVN-FL
```

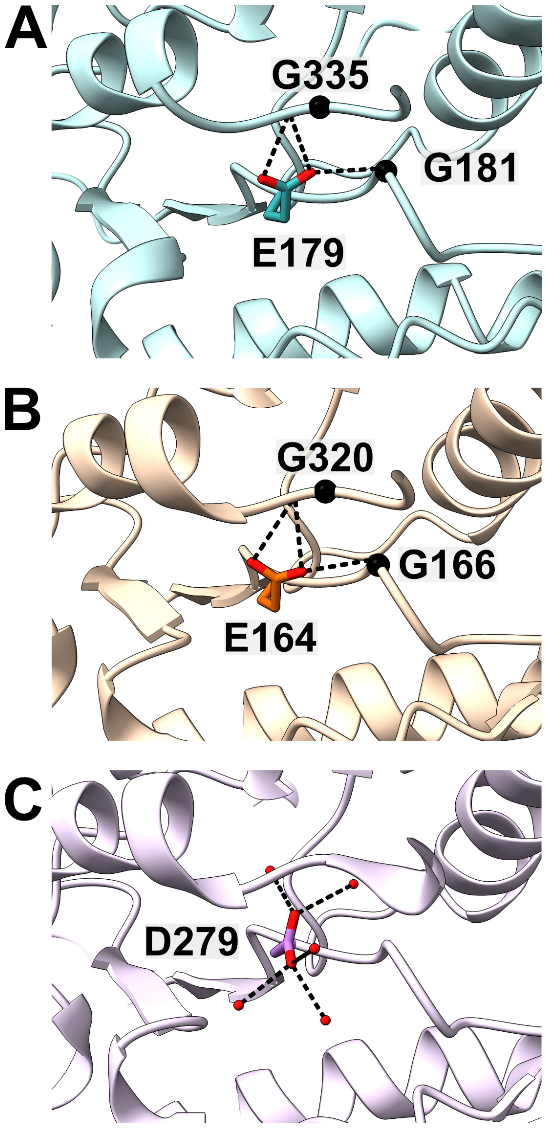

Figure S4: Interactions sustained by the negatively charged residue in the E/DxG motif

A. ELG motif in medaka titin kinase; **B.** EFG in human titin kinase; **C.** DFG in twitchin kinase.

References

Fryer CJ, White JB, Jones KA. (2004). Mastermind recruits CycC:CDK8 to phosphorylate the Notch ICD and coordinate activation with turnover. Mol. Cell, 16(4):509–520.

Matsunaga Y, Qadota H, Ghazal N, Lesanpezeshki L, Dorendorf T, Moody JC, Ahier A, Matheny CJ, Vanapalli SA, Zuryn S, Mayans O, Kwong JQ, Benian GM. (2024). Protein kinase 2 of the giant sarcomeric protein UNC-89 regulates mitochondrial morphology and function. Comm. Biol., 7(1):1342.

Mirdita M, Schütze K, Moriwaki Y, Heo L, Ovchinnikov S, Steinegger M. (2022). ColabFold: Making protein folding accessible to all. Nature Methods, 19(6):679–682.
